# BDNF and IL-33 Dynamics in an Ultrasound Stress Model of Fibromyalgia-like Phenotypes

**DOI:** 10.3390/ijms27094051

**Published:** 2026-04-30

**Authors:** Careen A. Schroeter, Dmitrii Pavlov, Johannes P. M. de Munter, Alexei Umriukhin, Raymond Cespuglio, Maria Kuznetsova, Alexey V. Deykin, Sholpan Askarova, Michael Sicker, Anna Gorlova, Tatyana Strekalova

**Affiliations:** 1 Preventive & Environmental Medicine Unit, Clinic Kastanienhof, Statthalterhofweg 70, 50858 Köln-Junkersdorf, Germany; careen.schroeter@web.de; 2 Hotchkiss Brain Institute, University of Calgary, Calgary, AB T2N 1N4, Canada; mitchellalexpavlov@gmail.com; 3 Department of Psychiatry and Neuropsychology, Maastricht University, Universiteitsinsel 50, 6229 ER Maastricht, The Netherlands; h.demunter@neuroplast.com (J.P.M.d.M.);; 4 Department of Normal Physiology, Sechenov University, 117198 Moscow, Russia; alum1@yandex.ru (A.U.); kuznetsova_m_a3@student.sechenov.ru (M.K.); shaskarova@nu.edu.kz (S.A.); 5 Neuroscience Research Center of Lyon, ENES Team, Claude-Bernard Lyon-1 University, 69675 Bron, France; raymond.cespuglio@univ-lyon1.fr; 6 Laboratory of Genetic Technology and Gene Editing for Biomedicine and Veterinary, National Research Belgorod State University, 308015 Belgorod, Russia; alexei@deikin.ru; 7 Center for Life Sciences, National Laboratory Astana, Nazarbayev University, Astana 010000, Kazakhstan; 8 Rehabilitationsfachkliniken, Hochwald-Kliniken, 66709 Weiskirchen, Germany or dr-sickerweb.de; 9 Research and Education Resource Center, Peoples Friendship University of Russia (RUDN University), 117198 Moscow, Russia; 10 Division of Molecular Psychiatry, Center of Mental Health, University of Hospital Würzburg, Magarete-Höppel-Platz 1, 97080 Würzburg, Germany

**Keywords:** fibromyalgia, major depression, pain sensitivity, allodynia, ultrasound stress, aggression, brain-derived neurotrophic factor (BDNF), interleukin-33 (IL-33), animal model

## Abstract

Fibromyalgia, a syndrome characterized by hyperalgesia and ‘negative emotionality’, and major depressive disorder (MDD) demonstrate substantial overlaps in clinical, neurobiological, and therapeutic domains. Currently, treatment options for fibromyalgia remain limited; however, the epidemiology of this syndrome continues to grow worldwide. The use of animal models is indispensable for developing new treatment strategies for fibromyalgia. Meanwhile, the choice of animal paradigms is limited. Here, we used the ultrasound exposure of emotional stress on CBA, BALB/c, and C57BL/6 mouse strains to model this condition and to identify new molecular targets of fibromyalgia treatment. We exposed young male mice of three common strains to a three-week ultrasound stress (US) comprising emotionally negative and neutral frequencies of 20–25 kHz and 25–45 kHz, resulting in the development of altered pain sensitivity and signs of ‘negative emotionality’. Specifically, mice were studied for timid-like/aggressive behaviors and the tail flick response. Serum levels of corticosterone, cortisol, β-Endorphin, and brain-derived neurotrophic factor (BDNF), as well as brain gene expression of interleukin-33 (*Il-33*), *Bdnf*, and its receptor *Trkb* were investigated. Among the stressed mouse strains, C57BL/6 mice displayed augmented pain sensitivity, allodynia, and suppressed dominant behavior, whereas CBA and BALB/c mice demonstrated opposing changes. Glucocorticoid levels were increased in all stressed groups. Stressed C57BL/6 mice showed downregulated gene and protein expression of functionally inter-related BDNF and IL-33 molecules in the hippocampus, amygdala, and striatum, significantly correlating with behavioral outcomes, as well as lowered blood levels of β-Endorphin and elevated cortisol concentrations. Altogether, our study identified the BDNF/IL-33 regulatory pathway as a molecular correlate of fibromyalgia, and the use of US-exposed young C57BL/6 mice as a potential model that recapitulates this syndrome.

## 1. Introduction

Fibromyalgia is a chronic pain condition associated with persistent widespread pain and psychological distress, maladaptive cognitive–affective responses, and compromised central pain modulation [1,2]. According to DSM-5-TR, fibromyalgia is most closely aligned with Somatic Symptom Disorder (SSD) and characterized by altered pain processing, disproportionate distress, affective and stress-related psychopathology, and ‘negative emotionality’ [3,4]. Fibromyalgia frequently results in lower quality of life and greater healthcare utilization, particularly when comorbid with other conditions [5]. Specifically, fibromyalgia has high comorbidity with major depressive disorder (MDD), generalized anxiety disorder, post-traumatic stress disorder, and related neuropsychiatric conditions [6,7], which in 80% of cases precede fibromyalgia by approximately one year [4,5,6,7,8].

Fibromyalgia has a particularly high overlap with symptoms of MDD and pharmacotherapy, and it is the main therapeutic approach for both of them and is based on the use of classical antidepressants [9,10,11]. Between 25% and 65% of fibromyalgia patients meet the criteria for MDD, and 38% of patients with MDD meet the fibromyalgia criteria [12,13]. These two conditions share such overlapping symptoms, such as hyperalgesia, weakness, tiredness, anhedonia, and affective symptoms, including anger, irritability, and anxiety [14,15]. Proposed mechanisms for this overlap include dysregulation of hypothalamic–pituitary–adrenal axis (HPA) function, impaired noradrenergic and serotonergic regulation, inflammatory processes involving cytokines, such as IL-6 and IL-8, and deficient pain inhibition [4,16].

Recently, a ‘top-down’ concept of fibromyalgia linked to psychological stress has been proposed, suggesting that behavioral alterations contribute to the main fibromyalgia symptoms through neuroendocrine stress mechanisms [17]. These behavioral stress-induced abnormalities may include over-aggressiveness [18,19] or, on a contrary, a ‘timid’ behavior and helplessness [20,21]. A strong correlation between stress-induced negative emotions and pain scores was recently reported [14,22,23]. As such, these behavioral symptoms of MDD and fibromyalgia may have a common neurobiological basis and a target of therapy.

Both disorders are considered to share underlying etiology, such as emotional (psychological) stress [14,15,24,25]. This form of stress is a state that is primarily triggered by the perception and cognitive evaluation of adverse events rather than a disturbance of physical nature [26,27,28], is an important etiological contributor of MDD [29,30,31] and fibromyalgia symptoms [32,33]. As such, neurobiological mechanisms of these diseases are associated with compromised stress regulation, including dysregulation of the HPA axis [34]. For fibromyalgia, additional involvement of neuropeptides, including substance P, abnormalities in central and peripheral serotonergic and noradrenergic transmission, and neuroinflammation, were shown [35,36]. Several mechanisms were shown to lead to a key pathophysiological feature of fibromyalgia, an impaired endogenous pain inhibition that is due to compromised pain processing, leading to hyperalgesia [35,37,38]. However, current knowledge about these mechanisms remains scarce, and molecular pathways underlying specific symptoms of fibromyalgia remain poorly understood. Meanwhile, a better understanding of molecular changes that underpin this disorder is critical for effective management of this epidemiologically growing condition [36,39,40].

Long-term fibromyalgia management integrates various treatment options, including pharmacological and non-pharmacological approaches, such as antidepressant medications [41,42,43], cannabinoid-based compositions, nonsteroidal anti-inflammatory drugs, and NMDA receptor antagonists [44]. Acupuncture, cognitive therapy, and exercise [43] remain costly methods of fibromyalgia management [45]. Currently, fibromyalgia treatment is considered to remain expensive, time-consuming, and when pharmacological treatments are applied, risky in terms of adverse effects of hepatic damage, cardiovascular problems, and weight gain [43,46]. This necessitates the search for more specific new compounds with potentially fewer adverse effects for treating fibromyalgia.

Animal models are indispensable in the studies of the mechanisms of fibromyalgia and identification of new pharmacological therapies; however, modeling this syndrome is considered to be highly challenging [47]. Various methods that are described in the literature often lack etiological relevance, face validity, and have other problems [48,49]. Many of them use repeated muscle damage, biogenic amine depletion, and physical stressors, such as intermittent cold stress [50,51]. The latter paradigm, one of the commonly used fibromyalgia models, was shown to induce mechanical and thermal allodynia, hyperalgesia, and behavioral aberrations in cognition and emotionality [51]. However, most animal models of fibromyalgia fail to employ etiologically relevant stressors and replicate negative emotions that parallel fibromyalgia in a clinic [50]. On the other hand, reported animal paradigms aiming to model MDD, overlapping with the fibromyalgia condition, using emotional stress in rodents, do not address the features of fibromyalgia and address its neurobiology [52,53,54,55].

Therefore, in the current study, we chose to employ a recently established model of emotional stress, in which mice following a three-week ultrasound stress (US) comprising emotionally negative and neutral frequencies of 20–25 kHz and 25–45 kHz, respectively, develop depressive-like syndrome manifested by behavioral signs of negative emotionality and MDD-like molecular, hormonal, and biochemical aberrations [56,57,58,59,60,61]. In this paradigm, mice are subjected to variable ultrasonic frequencies that are naturally emitted by rodents at their state of fear and anxiety, recapitulating human ‘psychological stress’ and thus omitting physical stressors [62,63,64]. US-exposed mice display helplessness, decreased sensitivity to reward, and altered social behavior and cognition, as well as increased blood CORT concentration, reduced expression of hippocampal neuroplasticity markers postsynaptic density protein 95 (PSD-95), polysialylated neural cell adhesion molecule (PSA-NCAM), β-catenin, cell proliferation, compromised glutamatergic and serotoninergic regulation, elevated oxidative stress markers malondialdehyde, protein carbonyl, and total glutathione in the brain, and brain upregulation of pro-inflammatory cytokines IL-1β, IL-6, and TNF [56,61,65]. Most of these changes were counteracted by a chronic administration of classic antidepressants and anti-oxidant treatments [56,58,59,60,62].

Previous studies showed that chronic US exposure induced aggressive behavior in male BALB/c mice [56,65] and suppressed social dominance in the C57BL/6 strain [61,64]. Given high prevalence of fibromyalgia in both sexes [66] and revealed inter-strain differences in manifestations of ‘negative behaviors’ in the US-exposed mice, and the importance of adequate selection of laboratory strain for pre-clinical research, we chose to compare three of the most commonly used male mice of inbred strains of laboratory mice: C57BL/6, BALB/c, and CBA mice [67,68]. While these lines are broadly used in neurobiological studies, they were shown to display distinct baseline and stress-induced behaviors in tests for locomotion [69,70], helplessness [71], exploration, and spatial learning [68,72,73]. Because large cohort studies have shown that the symptoms of fibromyalgia and comorbid neuropsychiatric abnormalities are similar between the sexes [74,75], and given the significant cycle-related constraints in using female animals in pain and emotionality assays, we chose to perform our exploratory study comparing three strains of male mice. In addition, some studies have shown greater rates of neuropsychiatric symptoms in male patients with comorbid fibromyalgia syndrome [66]. The importance of psychological aspects of fibromyalgia in females in comparison to males makes better face validity modeling more achievable in males [76].

Here, young male mice in three strains were exposed to the 3-week US and studied for ‘emotionally negative’ behavior, i.e., timid-like behavior/aggression, in the resident–intruder and social interaction paradigms, as well as in the pain sensitivity tail flick test. In an additional experiment, C57BL/6 male mice were studied using the von Frey filament test, Randall–Selitto test, and acetone evaporation test, as well as ELISA assay of several molecular markers potentially related to fibromyalgia. As we found in US-exposed mice displaying a common pattern of a suppression of dominant behavior in social interactions, low social exploration, and submissive social traits in interactions with aggressive counter-partners, this pattern was considered as ‘timid-like behavior’ that parallels the human construct ‘timid behavior’ [77,78], maladaptive behavior associated with clinical fibromyalgia [79,80,81]. The choice of tests used was based on the fact that hyperalgesia is a key symptom of fibromyalgia [1], which can be aggravated by emotional stress and is associated with aggression [82], as altered pain sensitivity may affect social behaviors [83,84,85]. Thereafter, blood and brain tissues were examined for corticosterone and cortisol levels and selected molecular markers associated with stress, aggression, and pain sensitivity [86]. In addition, we measured the blood plasma concentration of BDNF and β-Endorphin to assess the hormonal regulation of pain [87].

Specifically, in this study, we chose to test the hypothesis about the role of inter-related central expression of brain-derived neurotrophic factor (BDNF) and interleukin-33 (IL-33) in the mechanisms of fibromyalgia and emotional regulation. Central effects of BDNF that, along with its receptor TrkB in the amygdala [88], hippocampus [89], and the striatum [90], regulate aggression were shown to depend on the expression of IL-33, a pro-inflammatory cytokine that is involved in chronic pain and inflammation [91,92,93]. Recently, the attention of researchers was attracted to IL-33, which is a member of the IL-1 family, playing an important role in allergic reactions, chronic inflammation, and rheumatic immune diseases, on one hand [91], and the NF-κB-mediated regulation of BDNF expression [94], and thus mechanisms of ‘negative emotionality’ and social behavior, on the other hand. For example, a recent study demonstrated the BDNF mRNA overexpression following peripheral IL-33 administration in a rat model of sciatic nerve crush [95]. The role of BDNF in the regulation of aggression was well documented in the experiments using BDNF knockout mice [96], overexpressing *Bdnf* AB-Gatersleben inbred animals [97], and i.c.v. BDNF administration [98]. Notably, the manipulation of BDNF levels in the brain resulted in various outcomes depending on background strain, e.g., C57BL/6 or CBA [96,98].

IL-33 can be released in response to cellular stress or damage and acts as an alarmin cytokine, linking tissue perturbation and sterile inflammation to neuroimmune activation [99,100,101]. Through its receptor ST2, IL-33 activates intracellular pathways, such as MyD88, NF-κB, and MAPK, which are known to promote microglial activation, cytokine production, and glia–neuron communication in pain processing regions of the central nervous system [100,102]. Upregulation of IL-33/ST2 signaling has been demonstrated in experimental models of inflammatory and neuropathic pain; genetic or pharmacological inhibition of this pathway attenuates mechanical hyperalgesia and allodynia [103,104]. Hence, the evidence implicating BDNF/IL-33 regulation in the mechanisms of chronic pain, aggression, and inflammation led to hypothesizing the role of this pathway in fibromyalgia.

Here, gene and protein expression of IL-33, BDNF, and TrkB were assessed in the limbic brain structures, whose role is well documented, apart from the above-discussed aggressive modulation in the neurobiology of fibromyalgia, as was shown for the amygdala [105], the hippocampus [106], and striatum [107].

## 2. Results

### 2.1. Ultrasound-Induced Alterations in Scores of Aggression and Pain Sensitivity and Their Correlations in Different Mouse Strains

**Figure 1:**
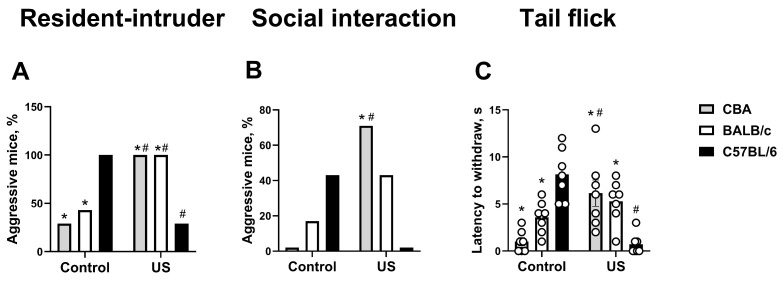
Evaluated parameters of aggressive behavior and pain sensitivity. (**A**) Percentage of mice that demonstrated aggressive behavior in the resident–intruder test. (**B**) Percentage of aggressive animals in the social interaction test. (**C**) Latency to withdraw the tail in the tail flick test. * *p* < 0.05 vs. non-stressed or stressed C57BL/6 strain, # *p* < 0.05 vs. the respective non-stressed strain. Two-way ANOVA and post hoc Tukey’s test, chi-square test; all groups are *n* = 7. US—ultrasound. All data are mean ± SEM.

The percent of mice that demonstrated aggressive behavior in the resident–intruder test was significantly higher in non-stressed C57BL/6 mice than in non-stressed CBA mice (*p* = 0.0053, chi-square test) and non-stressed BALB/c mice (*p* = 0.018). This measure was significantly increased in stressed CBA mice (*p* = 0.0053) and BALB/c mice (*p* = 0.018) and decreased in stressed C57BL/6 mice (*p* = 0.0053) compared to the respective non-stressed strains. Stressed C57BL/6 mice had a lower percentage of aggressors in comparison with stressed CBA mice and BALB/c mice (both *p* = 0.0053; Figure 1A). The number of aggressive animals in the social interaction test was significantly higher in stressed CBA mice compared to non-stressed CBA mice and stressed C57BL/6 mice (both *p* = 0.0053, chi-square test; Figure 1B). A strong trend for an increase in this measure was found in non-stressed C57BL/6 mice compared to non-stressed CBA mice (*p* = 0.0507). Stressed C57BL/6 mice had a trend for a decrease in this parameter in comparison with both non-stressed C57BL/6 mice and stressed BALB/c mice (both *p* = 0.0507).

ANOVA revealed significant stress × genotype interaction (F_(2,36)_ = 27.67, *p* < 0.0001, two-way ANOVA), but there was no significant stress or genotype effects (F_(1,36)_ = 0.07, *p* = 0.791 and F_(2,36)_ = 0.64, *p* = 0.532, respectively) for latency to withdraw the tail in the tail flick test. Between non-stressed mice, this measure was significantly higher in C57BL/6 mice in comparison with both CBA mice (*p* < 0.0001, Tukey’s test) and BALB/c mice (*p* = 0.0086). It was significantly increased in stressed CBA mice (*p* = 0.0024) and reduced in stressed C57BL/6 mice (*p* < 0.0001) compared to the respective non-stressed groups. Stressed C57BL/6 mice had this parameter significantly lower than stressed CBA mice (*p* = 0.0012) or stressed BALB/c mice (*p* = 0.0086; Figure 1C).

**Figure 2:**
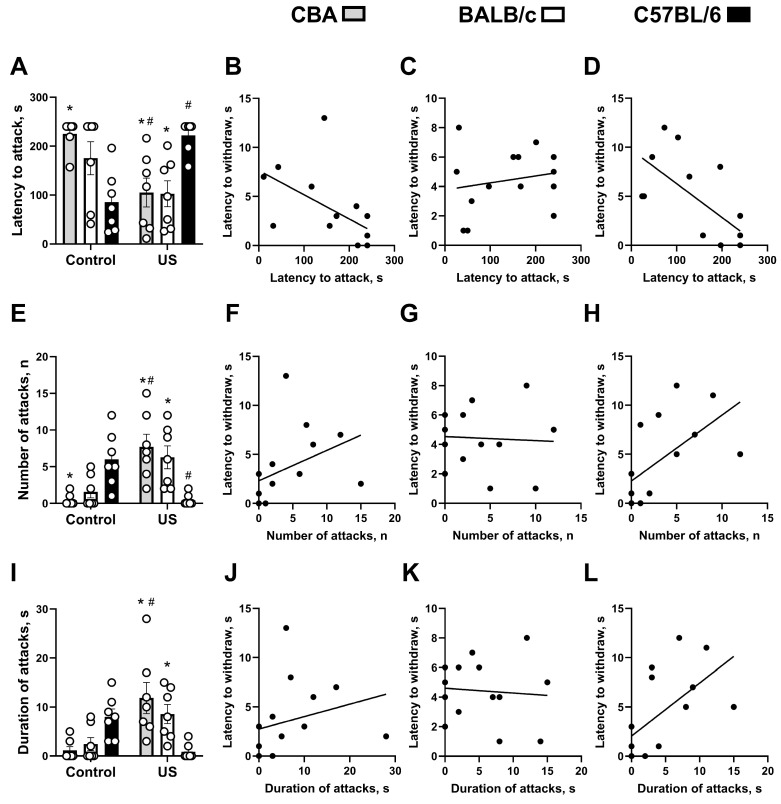
Effects of chronic ultrasound exposure on parameters of aggressive behavior in tests and their correlations with pain sensitivity. (**A**) Latency to attack in the resident–intruder test and (**B**) its correlation with latency to withdraw the tail in CBA mice and (**C**) BALB/c mice, and (**D**) a significant negative correlation was found between latency to attack and latency to withdraw the tail in the tail flick test in C57BL/6 mice. (**E**) Number of attacks in the resident–intruder test and (**F**) its correlation with latency to withdraw the tail in CBA mice, (**G**) BALB/c mice, and (**H**) C57BL/6 mice. (**I**) Duration of attacks in the resident–intruder test and (**J**) its correlation with latency to withdraw the tail in CBA mice, (**K**) BALB/c mice, and (**L**) C57BL/6 mice. * *p* < 0.05 vs. non-stressed or stressed C57BL/6 strain, # *p* < 0.05 vs. respective non-stressed strain. Two-way ANOVA and post hoc Tukey’s test, Spearman’s correlation, all *n* = 7. US—ultrasound. All data are mean ± SEM.

Significant stress × genotype interaction (F_(2,36)_ = 15.73, *p* < 0.0001, two-way ANOVA), but no significant stress or genotype effects (F_(1,36)_ = 0.88, *p* = 0. 354 and F_(2,36)_ = 0.582, *p* = 0.563, respectively), were revealed for latency to attack in the resident–intruder test. In non-stressed mice, it was significantly shorter in C57BL/6 but not in BALC/c mice than in CBA mice (*p* = 0.0032 and *p* = 0.1213, respectively, Tukey’s test). This parameter was significantly reduced in stressed CBA mice (*p* = 0.154) and elevated in C57BL/6 mice (*p* = 0.0041) compared to the respective non-stressed strains. Among the stressed groups, C57BL/6 mice demonstrated significantly longer latency to attack compared to both CBA mice (*p* = 0.193) and BALB/c mice (0.0159; Figure 2A). In CBA and C57BL/6 mice, significant negative correlations were found between this measure and the latency to withdraw the tail in the tail flick test (τ-b = −0.518, *p* = 0.015 and τ-b = −0.512, *p* = 0.017, respectively; Figure 2B,D), but no significant correlation was found in BALB/c mice (τ-b = +0.107, *p* = 0.613; Figure 2C).

ANOVA showed a significant stress effect (F_(1,36)_ = 5.105, *p* = 0.03, two-way ANOVA) and its interaction with the genotype (F_(2,36)_ = 17.15, *p* < 0.0001), but there was no significant genotype effect (F_(2,36)_ = 0.312, *p* = 0.734) for the number of attacks in the resident–intruder test. It was significantly higher in non-stressed C57BL/6 but not BALB/c mice than in non-stressed CBA mice (*p* = 0.0195 and *p* = 0.1109, respectively, Tukey’s test). Stressed C57BL/6 mice had this measure significantly decreased (*p* = 0.0195), while stressed CBA mice, on the contrary, were elevated (*p* = 0.0011) when compared to the respective non-stressed groups. Stressed C57BL/6 mice had a significantly lower number of attacks than CBA (*p* = 0.011) and BALB/c mice (0.0124; Figure 2E). In CBA and C57BL/6 mice, significant positive correlations were found between this parameter and latency to withdraw the tail (τ-b = +0.485, *p* = 0.023 and τ-b = +0.543, *p* = 0.012, respectively; Figure 2F,H); no significant correlation was found in BALB/c mice (τ-b = −0.012, *p* = 0.955; Figure 2G).

A significant stress effect (F_(1,36)_ = 4.88, *p* = 0.0336, two-way ANOVA) and a significant stress × genotype interaction (F_(2,36)_ = 13.35, *p* < 0.0001), but no significant genotype effect (F_(2,36)_ = 0.666, *p* = 0.519), were shown for the total duration of attacks in the resident–intruder test. Non-stressed BALB/c mice did not differ from the non-stressed C57BL/6 group in this parameter (*p* = 0.2651, Tukey’s test), while CBA mice showed a strong trend for such differences (*p* = 0.0998). At the same time, stressed CBA mice displayed significantly longer duration of attacks compared to non-stressed CBA mice (*p* = 0.02), as well as to stressed C57BL/6 mice (*p* = 0.0015). The latter also had significantly shorter attack durations than stressed BALB/c mice (*p* = 0.0466; Figure 2I), and there was a strong trend for its decrease compared to the non-stressed C57BL/6 group (*p* = 0.0781). Significant positive correlations were found between this indicator and latency to withdraw the tail in CBA (τ-b = +0.437, *p* = 0.041; Figure 2J) and C57BL/6 mice (τ-b = +0.503, *p* = 0.019; Figure 2L), but not in BALB/c mice (τ-b = −0.036, *p* = 0.866; Figure 2K).

**Figure 3:**
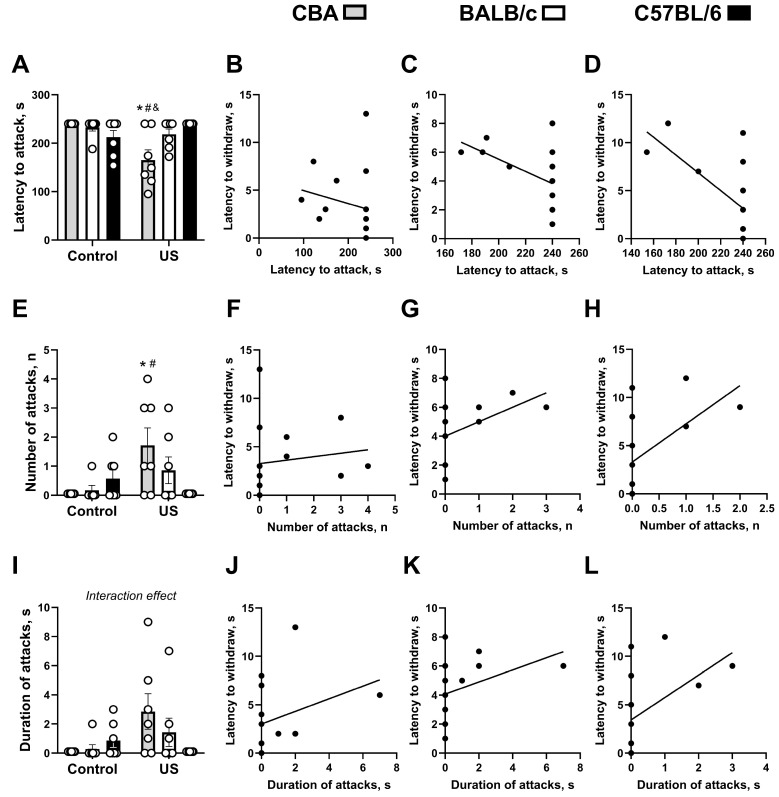
Effects of chronic ultrasound exposure on parameters of aggressive behavior. Effects of chronic US exposure on parameters of aggressive behavior in the social interaction test and their correlations with pain sensitivity. (**A**) Latency to attack in the social interaction test and (**B**) its correlation with latency to withdraw the tail in CBA mice, (**C**) BALB/c mice, and (**D**) C57BL/6 mice. (**E**) Number of attacks in the social interaction test and (**F**) its correlation with latency to withdraw the tail in CBA mice, (**G**) BALB/c mice, and (**H**) C57BL/6 mice. (**I**) Duration of attacks in the social interaction test and (**J**) its correlation with latency to withdraw the tail in CBA mice, (**K**) BALB/c mice, and (**L**) C57BL/6 mice. * *p* < 0.05 vs. stressed C57BL/6 strain, # *p* < 0.05 vs. respective non-stressed strain, & *p* < 0.05 vs. stressed BALB/c strain. Two-way ANOVA and post hoc Tukey’s test, Spearman’s correlation, all *n* = 7. US—ultrasound. All data are mean ± SEM.

A significant stress effect (F_(1,36)_ = 4.56, *p* = 0.0396, two-way ANOVA) and a stress × genotype interaction (F_(2,36)_ = 9.69, *p* = 0.0004), as well as a strong trend for the genotype effect (F_(2,36)_ = 2.67, *p* = 0.0832), were revealed for latency to attack in the social interaction test. Stressed CBA mice showed significantly decreased latency to attack the juvenile male in comparison with non-stressed CBA mice (*p* = 0.0008, Tukey’s test) and both stressed BALB/c mice (*p* = 0.284) and C57BL/6 mice (*p* = 0.0008; Figure 3A). No other significant group differences were revealed (*p* > 0.05). The latency to withdraw the tail correlated negatively with the latency to attack in the social interaction test in BALB/c mice (τ-b = −0.453, *p* = 0.050; Figure 3C) and C57BL/6 mice (τ-b = −0.512, *p* = 0.029; Figure 3D), but not in CBA mice (τ-b = −0.336, *p* = 0.136; Figure 3B).

ANOVA revealed a significant stress effect (F_(1,36)_ = 4.63, *p* = 0.0384, two-way ANOVA) and its interaction with the genotype (F_(2,36)_ = 5.57, *p* = 0.008), but there was no significant genotype effect (F_(2,36)_ = 1.4, *p* = 0.289) for the number of attacks in the social interaction test. Stressed CBA mice had this measure significantly increased when compared to non-stressed CBA mice (*p* = 0.0138, Tukey’s test) and stressed C57BL/6 mice (*p* = 0.0138; Figure 3E); no other significant group differences were revealed (*p* > 0.05). This parameter correlated positively with the latency to withdraw the tail in C57BL/6 mice (τ-b = +0.501, *p* = 0.035; Figure 3H), with a strong trend in BALB/c mice (τ-b = +0.467, *p* = 0.056; Figure 3G) and no significant correlation in CBA mice (τ-b = +0.313, *p* = 0.171; Figure 3F).

A significant stress × genotype interaction was shown for the duration of attacks in the social interaction test (F_(2,36)_ = 3.79, *p* = 0.0321, two-way ANOVA); a strong trend for the stress effect was found (F_(1,36)_ = 3.61, *p* = 0.0654), but no significant genotype effect was revealed (F_(2,36)_ = 1.1, *p* = 0.342). Post hoc analysis did not show any significant differences (*p* > 0.05; Figure 3I). This indicator showed significant positive correlations with the latency to withdraw the tail in BALB/c mice (τ-b = +0.475, *p* = 0.042; Figure 3K) and C57BL/6 mice (τ-b = +0.476, *p* = 0.042; Figure 3J), but not in the CBA group (τ-b = +0.291, *p* = 0.208; Figure 3J).

### 2.2. Ultrasound-Induced Gene Expression Alterations in Three Mouse Strains

**Figure 4:**
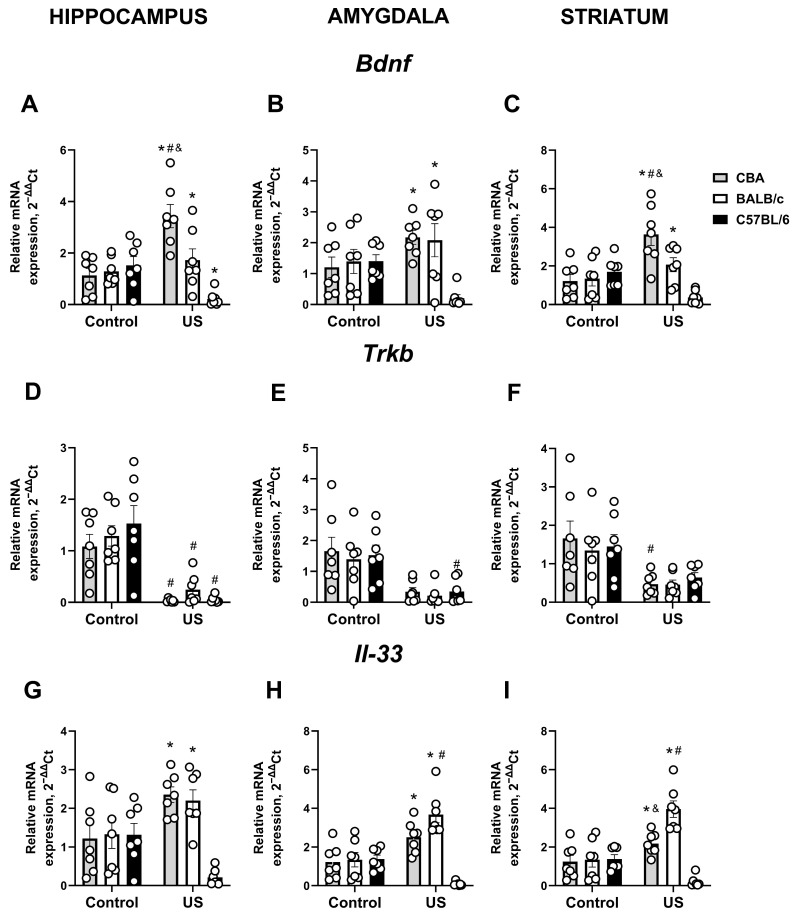
Effects of chronic ultrasound exposure on *Bdnf*/*Il-33* expression in inbred mouse strains. (**A**) *Bdnf* expression in the hippocampus, (**B**) amygdala, and (**C**) striatum. (**D**) *Trkb* expression in the hippocampus, (**E**) amygdala, and (**F**) striatum. (**G**) *Il-33* expression in the hippocampus (**H**,**I**) and striatum. * *p* < 0.05 vs. stressed C57BL/6 strain, # *p* < 0.05 vs. respective non-stressed strain, & *p* < 0.05 vs. stressed BALB/c strain. Two-way ANOVA and post hoc Tukey’s test, all *n* = 7. US—ultrasound. All data are mean ± SEM.

A significant stress × genotype interaction (F_(1,36)_ = 15.19, *p* < 0.0001, two-way ANOVA), as well as a significant genotype effect (F_(2,36)_ = 9.37, *p* = 0.0005) and a strong trend for a stress effect (F_(2,36)_ = 3.24, *p* = 0.0803), were found for *Bdnf* expression in the hippocampus. This parameter was significantly increased in stressed CBA mice compared to non-stressed CBA mice (*p* = 0.0002, Tukey’s test). *Bdnf* expression in the hippocampus of stressed CBA mice was higher than in stressed BALB/c mice (*p* = 0.0 107) and stressed C57BL/6 mice (*p* < 0.0001); it was also higher in stressed BALB/c mice compared to stressed C57BL/6 mice (*p* = 0.0365; Figure 4A). Stressed C57BL/6 mice had this measure significantly lower than non-stressed C57BL/6 animals (*p* = 0.0002). A significant stress × genotype interaction and a significant genotype effect (F_(2,36)_ = 6.313, *p* = 0.0045 and F_(2,36)_ = 5.048, *p* = 0.0117, respectively), but no significant stress effect (F_(1,36)_ = 0.326, *p* = 0.571), were revealed for *Bdnf* expression in the amygdala. It was significantly lower in stressed C57BL/6 mice compared to both stressed CBA mice (*p* = 0.0021) and stressed BALB/c mice (*p* = 0.0037; Figure 4B). No other significant group differences were revealed by post hoc analysis (*p* > 0.05). Similarly, ANOVA showed a significant stress × genotype interaction (F_(2,36)_ = 12.95, *p* < 0.0001), a significant genotype effect (F_(2,36)_ = 7.17, *p* = 0.0024), and a trend for the stress effect (F_(1,36)_ = 4.05, *p* = 0.0518) for *Bdnf* expression in the striatum. *Bdnf* expression in the striatum of stressed CBA mice was increased in comparison with non-stressed CBA mice (*p* = 0.0006, Tukey’s test), stressed BALB/c mice (*p* = 0.0495), stressed C57BL/6 mice (*p* < 0.0001), and BALB/c mice in comparison with stressed C57BL/6 mice (*p* = 0.0273; Figure 4C), with no other significant group differences shown by post hoc analysis (*p* > 0.05).

A significant stress effect (F_(1,36)_ = 56.44, *p* < 0.0001, two-way ANOVA) but no significant genotype effect or stress × genotype interaction (F_(2,36)_ = 0.86, *p* = 0.429 and F_(2,36)_ = 0.82, *p* = 0.447, respectively) was shown for *Trkb* expression in the hippocampus. This parameter was significantly decreased in stressed CBA, BALB/c, and C57BL/6 mice when compared to non-stressed respective strains (*p* = 0.006, *p* = 0.0068, and *p* < 0.0001, respectively, Tukey’s test; Figure 4D). For *Trkb* expression in amygdala, a significant stress effect was revealed (F_(1,36)_ = 28.05, *p* < 0.0001), but there was no significant genotype effect (F_(2,36)_ = 0.24, *p* = 0.785) or stress × genotype interaction (F_(2,36)_ = 0.039, *p* = 0.962). Specifically, it was significantly lower in stressed C57BL/6 mice compared to non-stressed C57BL/6 mice (*p* = 0.0255, Tukey’s test; Figure 4E), with a strong trend for such changes in stressed CBA and BALB/c mice (*p* = 0.0583 and *p* = 0.058, respectively). Similarly, in the striatum, there was a significant stress effect (F_(1,36)_ = 18.53, *p* = 0.0001), but no significant genotype effect (F_(2,36)_ = 0.21, *p* = 0.812) or stress × genotype interaction (F_(2,36)_ = 0.277, *p* = 0.759) was shown for *Trkb* expression. In stressed CBA mice, this parameter was significantly lower than in non-stressed CBA mice (*p* = 0.042, Tukey’s test; Figure 4F) but not in stressed BALB/c and C57BL/6 mice compared to the respective non-stressed groups (*p* = 0.2248 and *p* = 0.3172, respectively).

A significant genotype effect (F_(2,36)_ = 9.59, *p* = 0.0005, two-way ANOVA) and a stress × genotype interaction (F_(2,36)_ = 8.703, *p* = 0.0008) but no significant stress effect (F_(1,36)_ = 1.71, *p* = 0.199) were demonstrated for *Il-33* expression in the hippocampus. Among stressed mice, this measure was significantly lower in C57BL/6 mice than in CBA mice (*p* < 0.0001, Tukey’s test) and BALB/c mice (*p* = 0.0002; Figure 4G). A strong trend for a decrease in this parameter was shown for stressed C57BL/6 mice compared to the non-stressed group (*p* = 0.0702). ANOVA showed a significant stress and genotype effect, as well as their interaction (F_(1,36)_ = 9.63, *p* = 0.0037; F_(2,36)_ = 17.25, *p* < 0.0001 and F_(2,36)_ = 18.58, *p* < 0.0001, respectively) for *Il-33* expression in the amygdala, which was significantly increased in the amygdala of stressed BALB/c mice compared to non-stressed BALB/c mice (*p* < 0.0001, Tukey’s test). This parameter was significantly lower in stressed C57BL/6 mice compared to both stressed CBA and BALB/c mice (both *p* < 0.0001; Figure 4H). A strong trend for a decrease in this parameter was shown for stressed C57BL/6 mice compared to the non-stressed group (*p* = 0.0527). For *Il-33* expression in the striatum, a significant stress and genotype effect and a stress × genotype interaction (F_(1,36)_ = 10.31, *p* = 0.0028; F_(2,36)_ = 19.26, *p* < 0.0001, and F_(2,36)_ = 20.23, *p* < 0.0001, respectively) were demonstrated. Stressed BALB/c mice had this indicator significantly increased compared to non-stressed BALB/c mice (*p* < 0.0001, Tukey’s test). In stressed mice, this measure was lower in C57BL/6 mice compared to both CBA (*p* = 0.0005) and BALB/c mice (*p* < 0.0001); it was also lower in CBA mice compared to BALB/c mice (*p* = 0.0022; Figure 4I). A strong trend for a decrease in this parameter was shown for stressed C57BL/6 mice compared to the non-stressed group (*p* = 0.0784).

### 2.3. Ultrasound-Induced Changes in Blood Serum of the Three Mouse Strains

**Figure 5:**
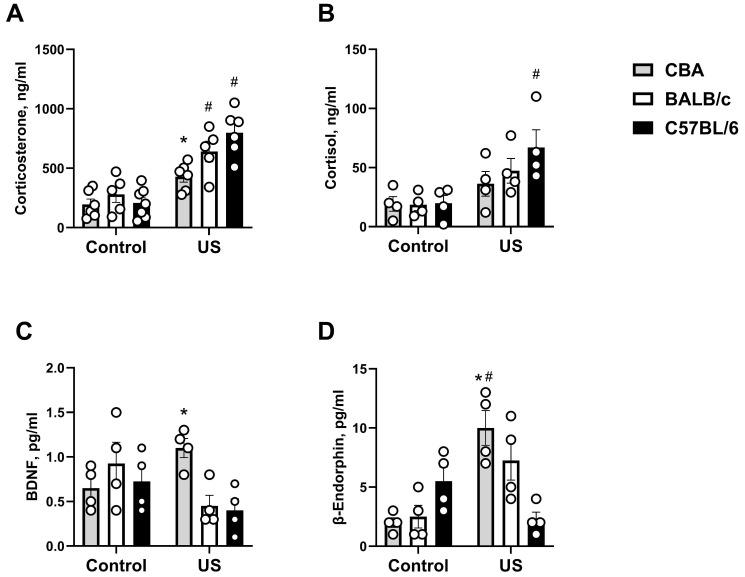
Effects of chronic ultrasound exposure on glucocorticoids, BDNF, and β-Endorphin in inbred mouse strains. (**A**) Corticosterone concentration. (**B**) Cortisol concentration. (**C**) BDNF concentration. (**D**) β-Endorphin concentration. * *p* < 0.05 vs. stressed C57BL/6 strain, # *p* < 0.05 vs. respective non-stressed strain. Two-way ANOVA and post hoc Tukey’s test, *n* = 5–7 for corticosterone analysis, for other measures, all *n* = 4. US—ultrasound. All data are mean ± SEM.

A significant stress effect, genotype effect, and their interaction were shown for serum corticosterone concentration (F_(1,36)_ = 60.15, *p* < 0.0001; F_(2,36)_ = 5.48, *p* = 0.0096, and F_(2,36)_ = 4.54, *p* = 0.0193, respectively, two-way ANOVA). This measure was significantly increased in stressed BALB/c mice (*p* = 0.008, Tukey’s test) and C57BL/6 mice (*p* < 0.0001) when compared to the respective non-stressed strains. It was also significantly higher in stressed C57BL/6 mice than in stressed CBA mice (*p* = 0.0024; Figure 5A). Stressed CBA mice did not differ significantly in this parameter from the non-stressed CBA group (*p* = 0.1029), and stressed BALB/c mice did not differ from stressed CBA mice (*p* = 0.2134) or stressed C57BL/6 mice (*p* = 0.5169). ANOVA revealed a significant stress effect (F_(1,36)_ = 15.86, *p* = 0.0009) but no significant genotype effect (F_(2,36)_ = 1.39, *p* = 0.273) or stress × genotype interaction (F_(2,36)_ = 1.28, *p* = 0.302) for serum cortisol concentration. In stressed CBA mice, it was significantly higher than in non-stressed CBA mice (*p* = 0.0259; Figure 5B). No other significant group differences were found (*p* > 0.05).

A significant stress × genotype interaction (F_(2,36)_ = 5.24, *p* = 0.0161, two-way ANOVA) but no significant stress or genotype effects were found (F_(1,36)_ = 0.86, *p* = 0.364 and F_(2,36)_ = 2.1, *p* = 0.151, respectively). In stressed CBA mice, this parameter was significantly higher than in stressed C57BL/6 mice (*p* = 0.0455, Tukey’s test; Figure 5C), and a strong trend for such a difference was found between stressed CBA and BALB/c mice (*p* = 0.0714). No other significant group differences were found (*p* > 0.05).

ANOVA demonstrated a significant stress effect, as well as a stress × genotype interaction (F_(1,36)_ = 11.58, *p* = 0.0032 and F_(2,36)_ = 12. 91, *p* = 0.0003, respectively, two-way ANOVA) but no genotype effect (F_(2,36)_ = 1.74, *p* = 0.204) for concentration of serum β-Endorphin. It was significantly higher in stressed CBA mice compared to non-stressed CBA mice (*p* = 0.0012, Tukey’s test) and stressed C57BL/6 mice (*p* = 0.0017; Figure 5D). Stressed BALB/c mice had a strong trend for such a difference compared to the non-stressed BALB/c group (*p* = 0.0782) but did not differ from the stressed C57BL/6 group (*p* = 0.577). Stressed C57BL/6 mice also did not differ significantly in this measure compared to the respective non-stressed group (*p* = 0.3713).

### 2.4. Ultrasound-Induced Increase in Allodynia and Brain Protein Changes in C57BL/6 Mice

**Figure 6:**
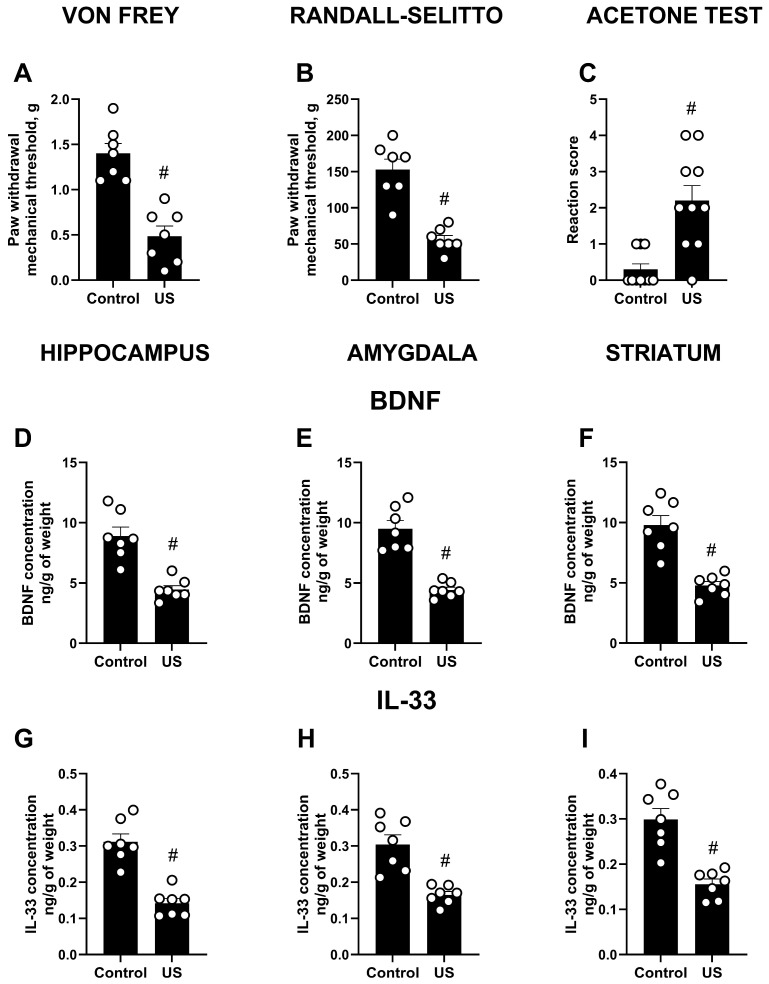
Results of pain sensitivity tests and brain ELISA in the C57BL/6 strain. (**A**) von Frey test, (**B**) Randall–Selitto test, (**C**) acetone evaporation test. (**D**) BDNF concentration in the hippocampus, (**E**) amygdala, and (**F**) striatum. (**G**) IL-33 concentration in the hippocampus (**H**,**I**) and striatum. # *p* < 0.05 vs. non-stressed C57BL/6 group, *t*-test, or Mann–Whitney test, *n* = 7–10. US—ultrasound. All data are mean ± SEM.

Stressed C57BL/6 mice had significantly lower paw withdrawal threshold in the von Frey test compared to the non-stressed group (*p* < 0.0001, *t*-test; Figure 6A). Similarly, the withdrawal threshold was significantly decreased in stressed C57BL/6 mice in comparison with the non-stressed C57BL/6 group in the Randall–Selitto test (*p* < 0.0001, *t*-test; Figure 6B). The number of withdrawal responses in the acetone test was significantly elevated in stressed C57BL/6 mice compared to the non-stressed group (*p* = 0.0012, Mann–Whitney test; Figure 6C).

BDNF concentration was significantly decreased in stressed C57BL/6 mice compared to the non-stressed group in the hippocampus, amygdala, and striatum (all *p* < 0.0001, *t*-test; Figure 6D–F). IL-33 concentration was significantly lower in the hippocampus (*p* < 0.0001, *t*-test; Figure 6G), amygdala (*p* = 0.0002, *t*-test; Figure 6H), and striatum (*p* = 0.0003, *t*-test; Figure 6I) of stressed C57BL/6 mice than in the non-stressed C57BL/6 group.

Correlative analysis revealed significant correlations between behavioral and molecular scores. All statistical values are presented in Supplementary Materials Tables S2–S4.

Table 1 summarizes shared correlations between different mouse strains.

## 3. Discussion

The association between increased pain sensitivity and ‘negative behavior’, often accompanied by MDD-like symptoms, is central to recapitulating the key features of fibromyalgia in rodent models of this syndrome. The present study showed that following ultrasound stress, among the most commonly utilized inbred mouse strains, CBA, BALB/c, and C57BL/6, male mice, the latter strain displayed timid-like behavior, hyperalgesia, and signs of allodynia, significantly correlating with the expression of BDNF receptor *Trkb* and *Il-33* in the limbic system, eminently augmented corticosterone and cortisol levels, and reduced concentrations of BDNF and β-Endorphin in blood. Notably, the observed alterations in gene expression were supported at the protein level in an experiment on male C57BL/6 mice, consistent with early observations for BDNF in this model [62]. Importantly, allodynia-like responses in the von Frey test, acetone evaporation assay, and Randall–Selitto of ultrasound-exposed C57BL/6 mice corroborated the tail flick test results, collectively indicating the development of hyperalgesia in these animals. A suppressed dominance and manifestations of timid-like behavior are well established as typical behavioral manifestations of MDD in males associated with an increased risk of fibromyalgia [81,84,108,109]. This finding, along with the demonstration of a pronounced hormonal stress response, allows us to consider the model of ‘emotional stress’ employed here in C57BL/6 mice as a promising paradigm for modeling this syndrome. Our results indirectly support the hypothesis that the central BDNF/IL-33 regulatory loop is a potential mechanism contributing to fibromyalgia.

Previous studies have extensively validated the ultrasound stress paradigm in male C57BL/6 mice and demonstrated the induction of MDD-like symptoms, such as anhedonia in the sucrose test, helplessness in the swim test and tail suspension paradigm, and cognitive impairment that were accompanied by anxiety-like behaviors in these mice [56,60,65]. An additional demonstration of hyperalgesia and allodynia in the current study suggests the validity of the ultrasound stress model in C57BL/6 mice in mimicking fibromyalgia-like syndrome. Other studies are in keeping with our findings, demonstrating that ten days of social defeat stress led to increased pain sensitivity in the hot plate test in C57BL/6J male mice [110].

The current study identified downregulated expression of the BDNF receptor *Trkb* in the hippocampus and amygdala of stressed C57BL/6 mice, which is in line with previous findings [62,63]. These changes were significantly correlated with the behavioral parameters in the resident–intruder and social interaction tests. Our study revealed significant correlations between brain *Trkb* expression and hyperalgesia, and the expression of this gene and social behavior, on the other hand, in all groups of stressed mice, suggesting its role in the regulation of pain sensitivity and dominant traits. However, as the reported downregulation of brain *Trkb* was found in all stressed groups, this does not allow for the suggestion of a specific involvement of *Trkb* in the fibromyalgia-like syndrome displayed by C57BL/6 mice. The similar downregulation of the BDNF receptor *TrkB* in all three mouse lines, regardless of the direction of changes in pain sensitivity and social behavior, further demonstrates the complexity of BDNF-related regulatory mechanisms in governing the development of this syndrome. Other studies support the role of Trkb in the regulation of aggressive behavior. For example, in aggressive Norway rats, reduced levels of the full-length TrkB (TrkB-FL) receptor were found, whereas the truncated TrkB (TrkB-T) form was increased, leading to a decreased TrkB-FL/TrkB-T ratio [111]. Male Long–Evans rats exposed to acute multiple stress [112] or repeated chronic mild stress [113] exhibited impaired BDNF-stimulated TrkB activation in the hippocampus, and acute immobilization stress in male Sprague–Dawley rats significantly increased BDNF mRNA and protein levels but reduced *Trkb* mRNA expression in the pituitary gland [114].

In contrast to other strains, stressed C57BL/6 mice displayed lower *Bdnf* gene expression, which is consistent with clinical findings regarding the role of BDNF in fibromyalgia [115]. Interestingly, BDNF blood levels were unchanged in stressed BALB/c mice that displayed a pronounced increase in aggression scores and were decreased in two other stress-exposed strains. This might suggest the role of central, rather than peripheral, BDNF changes in the development of the fibromyalgia-like syndrome in the employed paradigm. In addition, we revealed downregulated expression of *Il-33* in the amygdala of ultrasound-exposed groups, which also correlated with changes in social interactions in ultrasound-exposed C57BL/6 mice that displayed reduced social dominance. Previous studies have suggested that altered cytokine expression is characteristic of fibromyalgia [116]. A demonstration of suppressed expression of *Il-33,* being associated with timid-like behavior in C57BL/6 mice and elevated expression of this pro-inflammatory cytokine in other mouse strains showing sharply increased aggression scores, evidences the role of this molecule in the regulation of social behavior under stress conditions. The fact that these correlations were revealed in the amygdala, a well-established brain center regulating aggression, strengthens this conclusion.

A combination of emotional dysregulation and heightened pain sensitivity, associated with lowered β-Endorphin blood levels, is central to fibromyalgia [117,118]. Notably, our study revealed striking differences in β-Endorphin levels in the blood of stressed C57BL/6 mice, with a drastic decrease in this measurement compared to CBA and BALB/c male mice that had opposing changes. This finding may further support the validity of the investigated model as a paradigm of fibromyalgia. The changes in β-Endorphin blood levels in the three mouse lines were inter-related with BDNF concentrations in the blood, which is in keeping with previous reports in animal and clinical studies reporting similar results. Parallel changes in plasma β-Endorphin and BDNF levels were found in a rat model of neuropathic pain [119] and clinical studies in fibromyalgia patients [120,121], as well as in other clinical conditions [122], suggesting a coherent regulation of BDNF and β-Endorphin under hyperalgesia.

Interestingly, we found that CBA and BALB/c mice exhibited increased aggression following ultrasound exposure, showing more frequent and prolonged attacks towards unfamiliar opponents after chronic ultrasound exposure while also demonstrating reduced pain sensitivity. These changes were reciprocal with respect to the stress-induced effects in C57BL/6 mice.

The inter-strain differences reported in our study are consistent with well-established and reproducible phenomena in rodent research on strain differences in stress, emotionality, aggression, and pain-related phenotypes, reflecting the underlying role of genetic factors in these physiological processes [123,124,125,126]. Both hyperalgesia and hypoalgesia may result from stress and be symptoms of psychiatric disorders [127,128,129]. This is consistent with animal research showing that stress can produce either stress-induced hyperalgesia or stress-induced analgesia, depending on the stressor type, duration, predictability, and individual genetic predisposition [130,131,132,133]. Therefore, the fact that in the present study, a combination of genetic background and ultrasound stress exposure resulted in opposing effects in terms of changes in pain sensitivity, hormonal, and other measures, is not surprising. In addition, abundant studies show the role of genetic factors in the development of fibromyalgia [134].

Together, our study demonstrates that stress-induced alterations in pain sensitivity and social behavior can be inter-related, at least under stress conditions. This is further supported by the significant correlations of pain responses and social behaviors with the brain expression of the BDNF/IL-33 pathway in stressed male CBA and BALB/c mice. Reported changes in aggression and hypoalgesia were accompanied by elevated expression of *Bdnf* and *Il-33* in the hippocampus, amygdala, and striatum, brain regions implicated in pain modulation and emotional processing [135,136,137,138]. Moreover, the current study revealed elevated β-Endorphin concentrations in the blood plasma of stressed male CBA and BALB/c mice, whereas C57BL/6 mice exhibited the opposite change. Our findings on CBA and BALB/c mice align with previous studies demonstrating that Norway rats selectively bred for high aggression toward humans exhibit increased *Bdnf* mRNA and protein levels in the hippocampus, amygdala, and striatum compared to rats bred for lower aggression [137,139]. Stress-induced physiological effects are consistent with previous observations obtained in male BALB/c and C57BL/6 mice [56,65] and with other studies that compared C57BL/6 and CBA mice in social behavior and pain sensitivity [84,85]. Together, the accumulated evidence supports the view that adaptive pain inhibition mechanisms are linked to aggression [140].

Human and animal studies corroborate the association between pain sensitivity, altered social behavior, and fibromyalgia. Increased BDNF levels have also been associated with fibromyalgia syndrome. A meta-analysis found significantly higher BDNF levels in the blood of patients with fibromyalgia [115]. Duloxetine treatment significantly reduced serum BDNF levels, coinciding with improvement of pain and distress scores in patients with fibromyalgia [141]. In a fibromyalgia-like model in Wistar rats induced by reserpine injections, which are also known to result in depression-like symptoms [142], lower serum BDNF levels were associated with increased pain sensitivity in the hot plate test [143]. In addition, numerous other human studies have demonstrated an association between BDNF dysregulation, aggression, and pain sensitivity [144,145,146,147].

Notably, our study suggests the importance of IL-33, a pro-inflammatory cytokine whose role has previously been shown in immune responses [148] and regulation of *Bdnf* expression [97,149], but not in fibromyalgia syndrome. Other pro-inflammatory cytokines have been implicated in fibromyalgia symptoms. For instance, IL-6 and IL-8 have been reported to modulate symptom severity, with *Il-8* expression promoting sympathetic pain and *Il-6* expression inducing hyperalgesia, fatigue, and depression [150]. Our study suggests that *Il-33* expression in the brain may regulate pain sensitivity under stressful conditions and be associated with ‘timid-like’ behavior. Specifically, we showed that its downregulation was negatively correlated with aggressive behavior in stressed mice. Interestingly, previous studies on mutant mice lacking IL-33 on a BALB/c background revealed impaired social recognition in these animals [151].

The recent literature shows that IL-33 may represent an additional upstream regulator of inflammatory cascades, given its capacity to amplify cytokine production through the activation of NF-κB- and MAPK-dependent pathways [100,102]. This effect may be linked to IL-33-driven modulation of microglial activation and glial–neuronal communication, which can contribute to stress-induced neuroinflammation. Moreover, IL-33 influences synaptic plasticity and neuronal excitability, which may explain its association with altered behavior. These findings are consistent with emerging evidence of the role of neuroimmune signaling in the regulation of social behaviors under stress conditions [152,153,154].

Both corticosterone and cortisol concentrations in plasma were elevated in all ultrasound-exposed groups and were more pronounced in C57BL/6 mice, suggesting that this effect could be considered as an MDD-like feature [155] associated with altered social behaviors [156,157], as well as pain sensitivity [128,129]. Evidence suggests that glucocorticoid levels may positively correlate with pain sensitivity, as shown, for example, in a human study with male participants [158]. Another study showed that injections of corticosterone into the amygdala of male Fisher 344 rats increased pain sensitivity [159].

Our study demonstrated decreased pain sensitivity in aggressive CBA and BALB/c mice, further suggesting that under stress conditions, complex mechanisms may be involved in pain modulation. The exact molecular and neurobiological mechanisms underlying these strain-specific differences remain unclear and warrant further investigation using a broader range of molecular targets, which may be considered a limitation of this study. While we examined whether behavioral alterations in a mouse strain exhibiting elements of the fibromyalgia endophenotype were associated with hypothesized central regulatory mechanisms involving BDNF and IL-33, the study design does not allow conclusions regarding causality. Demonstrating a causal role of IL-33/BDNF signaling in this complex syndrome will require dedicated rescue experiments. In addition, the sample size used in this study was relatively small, which may limit the statistical power.

Another important limitation of this work is that it models only a subset of clinical forms of the disease, namely, fibromyalgia associated with MDD and male sex, given that female sex is associated with a higher prevalence of fibromyalgia [66]. Other forms of this syndrome, or the role of females and fibromyalgia, with leading symptoms of muscle pain that are problematic to assess in laboratory rodents, are likely not covered by the proposed paradigm. However, the goal of the present exploratory study was to perform a strain comparison and narrow down the general experimental conditions for recapitulating the fibromyalgia phenotype in mice. While the use of female mice is far more complicated to provide a sensible, controllable design, it is still possible and can be done in future experiments based on the predefined general experimental frame here.

## 4. Materials and Methods

### 4.1. Experimental Animals

The experiment was conducted using male CBA, BALB/c, and C57BL/6 mice (all *n* = 14), aged 3 months, obtained from the certified supplier Charles River (Janvier, Le Genest-Saint-Isle, France). Additionally, male 3-month-old CD1 mice (*n* = 42) from the same supplier were used as counter-partners in the resident–intruder test. For the social interaction test, juvenile CBA, BALB/c, and C57BL/6 mice (all *n* = 14), aged 3 weeks, were used with their respective strains. A separate cohort of male 3-month-old C57BL/6 mice (*n* = 20) from the same supplier was utilized for an additional ultrasound tress experiment to assess allodynia and to perform brain ELISA analysis. The total number of experimental mice was 62. Experimental mice were individually housed under standard laboratory conditions, with food and water available ad libitum. A reverse light/dark cycle was implemented, with lights on at 20:00 and off at 8:00. All behavioral experiments were conducted during the dark phase. Throughout the experimental period, animals were observed each morning and evening to monitor their condition. Potential confounding factors were controlled as described previously [160]. All protocols complied with Directive 2010/63/EU of 22 September 2010, ARRIVE guidelines. The study was granted approval from the Local Ethics committee at C. Bernard University (CBU 08RC2015, 3 August 2015). Every effort was made to minimize potential animal discomfort. The study did not have humane endpoints.

### 4.2. Study Flow

In each mouse strain, a stressed group (all *n* = 7) was subjected to a 21-day exposure to ultrasound with unpredictably alternating frequencies, as described elsewhere [56,63,64]. Sample size was determined based on previous studies employing this model, with the aim of balancing statistical power and ethical considerations [59,60,61]. The selected sample size was based on the power calculation for behavioral, hormonal, and molecular read-out studies in the ultrasound stress model, indicating that a sample size of seven animals per group provides sufficient power to detect the observed group differences at α = 0.05 [161]. It was consistent with previous studies’ design using the mouse ultrasound stress paradigm [56,65]. Randomization was performed according to body weight. Control groups (all *n* = 7) were housed separately in another laboratory room.

Following the ultrasound exposure, on day 22, all mice were investigated in a resident–intruder test. On day 23, a social interaction test was conducted using juvenile mice as counter-partners, followed by a tail flick test. On day 24, all mice were killed, and their hippocampus, amygdala, and striatum were extracted from dissected brains for RT-qPCR (reverse transcription quantitative real-time PCR) analysis. Additionally, blood samples were collected for ELISA assays.

**Figure 7:**
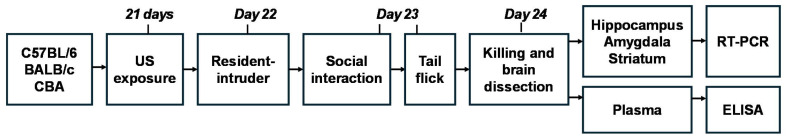
Study flow with CBA, BALB/c, and C57BL/6 mice. Mice were exposed to US stress for 21 days. Behavioral tests were conducted on days 22–23. On day 24, all animals were euthanized, and their brains were collected and dissected for subsequent analysis (see below).

In a separate experiment, C57BL/6 mice (*n* = 10) were subjected to a 21-day exposure to ultrasound, and the control group (*n* = 10) was not manipulated. Next, on day 21, seven mice per group were investigated in a von Frey test. On day 22, the Randall–Selitto test was conducted, and seven mice per group were used. On day 23, all mice were studied in the first session of the acetone test, and on the following day, the second session was performed, and the animals were killed. Their hippocampus, amygdala, and striatum were extracted from dissected brains for ELISA assays (Figure 7).

### 4.3. Ultrasound Stress

Chronic ultrasound exposure was carried out as previously described [56,57,58,60]. Over a 21-day period, experimental groups of mice were continuously exposed to ultrasound radiation at an average intensity of 50 ± 5 dB, with variable frequencies ranging from 20 to 45 kHz. The frequencies were alternated randomly every 10 min between 20–25 kHz, 25–40 kHz, and 40–45 kHz using a commercially available device (Weitech, Wavre, Belgium). The shape of the ultrasound signal was fluctuating, mimicking the natural ultrasonic vocalizations of mice [162]. The distribution of ultrasound radiation was controlled using an ultrasound detector (Discovery Channel, Rochester, NY, USA). The ultrasound device was suspended 2 m above the experimental cages, with an average horizontal distance of 2.5 m. To avoid positional bias, the cages were repositioned relative to the stimulator on a weekly basis. Previous studies demonstrated the selective adverse effects of low-frequency ultrasound, distinguishing them from potential general negative effects of constant white noise accompanying the procedure [62].

### 4.4. Resident–Intruder Test

A resident–intruder test was employed as previously described [56,65]. A previously group-housed naïve CD-1 male mice of similar age were used as intruders and introduced into the home cages of experimental mice for 8 min. Latency to attack, number, and total duration of attacks initiated by the residents were scored, as well as the number of mice that demonstrated aggressive behavior.

### 4.5. Social Interaction Test

In this test, juvenile male mice of the CBA, BALB/c, or C57BL/6 strain were introduced into the home cages of experimental mice for 8 min as previously described [163]. Latency to attack, number, and total duration of attacks initiated by the residents were scored, as well as the number of mice that demonstrated aggressive behavior.

### 4.6. Tail Flick Test

This test was used to evaluate pain sensitivity as described elsewhere [164]. After acclimatization to the testing environment, mice were placed in the restrained position with their exposed tail on a platform or within a holder. A halogen lamp was positioned above the tail, bright enough to cause noticeable discomfort but not intense enough to cause any physical harm. When the animal feels discomfort, it reacts with a sudden tail movement (tail flick), which automatically stops the stimulation and the timer for the measurement of the reaction time by the system used (Tail Flick Analgesia Meters, Panlab, Barcelona, Spain).

### 4.7. Von Frey Test

To assess mechanical sensitivity, mice were placed in individual compartments on an elevated wire mesh platform and allowed to acclimate for 10 min. A series of von Frey filaments of increasing force was applied perpendicularly to the plantar surface of the hind paw until the filament bent slightly, and it was held for 1 s. Withdrawal responses (e.g., paw lifting, shaking, or licking) were recorded. Mechanical thresholds were determined using the up–down method, whereby filament force was increased or decreased based on the presence or absence of a withdrawal response, and the 50% withdrawal threshold was calculated. Each paw was tested 3 times with 10 s between individual filament applications within a trial sequence and 2 min between repeated measurements of the same paw to avoid sensitization, and the average threshold was used for analysis.

### 4.8. Randall–Selitto Test

Deep tissue mechanical sensitivity was assessed in gently restrained mice, and a steadily increasing mechanical force was applied to the dorsal surface of the hind paw using a calibrated pressure device (Tail Flick Analgesia Meters, Panlab, Barcelona, Spain). Force was increased at a constant rate until a withdrawal response (e.g., paw withdrawal, struggle, or vocalization) was elicited, at which point the applied force was recorded as the withdrawal threshold. A predefined cutoff force of 200 g was used to prevent tissue injury. Each paw was tested 3 times at 15 min intervals to avoid sensitization, and the average threshold was used for analysis.

### 4.9. Cold Allodynia (Acetone Evaporation Test)

Cold allodynia was assessed using the acetone test, which evaluates nocifensive responses to mild cooling, a stimulus that is normally non-painful. The test is based on the rapid evaporation of acetone, producing a localized cooling effect on the skin. Mice were habituated for 30 min prior to testing in individual observation chambers (Techniplast, Rome, Italy) placed on an elevated metal mesh grid to minimize stress-related artifacts. A small drop of acetone (30 µL) was applied to the plantar surface of the hind paw using a pipette. Care was taken to avoid direct contact with the paw to prevent mechanical stimulation; the droplet was allowed to contact the skin via surface tension only. Behavioral responses were recorded for 30–60 s following application. Nocifensive behaviors included paw withdrawal, flicking or shaking, licking or biting, and prolonged guarding or lifting of the paw. Responses were quantified using a semi-quantitative scoring system: 0, no response; 1, brief withdrawal; 2, repeated flicking; and 3, licking or guarding behavior. Both hind paws were tested, with one paw assessed per day. The assay was repeated at 24 h intervals, and the average score per mouse was calculated from both measurements. All testing was conducted in a blinded manner.

### 4.10. Killing of Mice and Sample Collection

Mice were terminally anesthetized using CO_2_ and isoflurane, following the previously established protocols [165,166]. Blood was collected transcardially and stored in heparinized vials before centrifugation (1500 rcf, 15 min, 4 °C). The plasma was then separated and immediately stored at −80 °C until further use. Following blood collection, the mice were perfused with NaCl, after which the brain was extracted. The hippocampus, amygdala, and striatum were dissected, rapidly frozen on dry ice, and stored at −80 °C until needed.

### 4.11. Quantitative Real-Time PCR

Total mRNA was extracted from each sample using the RNeasy Lipid Tissue Mini Kit (Qiagen, Hilden, Germany). For first-strand cDNA synthesis, 1 μg of total RNA was reverse transcribed into cDNA using the QuantiTect Reverse Transcription Kit (Qiagen, Hilden, Germany) following the previously established protocols [165,166]. qRT-PCR was conducted with the SYBR Green master mix (Bio-Rad Laboratories, Philadelphia, PA, USA) on a ProFlex PCR system (Thermo Fisher Scientific, Waltham, MA, USA). Each reaction (10 μL) contained 5 μL of SYBR Green master mix, 3 μL of RNase-free water, 1 μL of specific forward and reverse primers (20 pmol/μL), and 1 μL of cDNA. Glyceraldehyde-3-phosphate dehydrogenase (*Gapdh*) was chosen as the reference gene due to its relatively stable expression in the brain, as previously reported [56,62]. The qRT-PCR protocol included an initial denaturation at 95 °C for 4 min, followed by 40 cycles of denaturation at 95 °C for 20 s and annealing at 54 °C for 90 s. Primer sequences are listed in Supplementary Materials Table S1. All samples were run in triplicate. Data were normalized to *Gapdh* mRNA expression and calculated as relative fold changes, following established method [56,60].

### 4.12. Enzyme-Linked Immunosorbent Assay (ELISA)

Commercially available Mouse BDNF ELISA Kit (Invitrogen, Carlsbad, CA, USA), Mouse Cortisol ELISA Kit (Lifespan Biosciences, Lynnwood, WA, USA), Mouse Corticosterone ELISA kit (Abcam, Cambridge, UK), and Mouse β-Endorphin ELISA kit (Antibodies, Cambridge, UK) were used to measure BDNF, cortisol, corticosterone, and β-Endorphin concentration, respectively. The optical densities of experimental plates were measured using a plate reader (Wallace 1420 VICTOR, Waltham, MA, USA). All samples were run in duplicate. All procedures were performed according to the instruction manual and carried out as previously described [63].

Protein levels of BDNF and IL-33 were measured using mouse ELISA kits (LS-F2404 and LS-F12907, respectively; LifeSpan Biosciences, Seattle, WA, USA) according to the manufacturer’s instructions. Samples were applied to antibody-precoated 96-well plates and incubated overnight at 4 °C with gentle shaking. After washing, biotinylated detection antibody was added and incubated for 1 h at room temperature, followed by incubation with streptavidin–horseradish peroxidase (HRP) conjugate for 45 min. Absorbance was measured at 450 nm using a microplate reader (FluoStar Optima, BMG Labtech, Cary, NC, USA), and protein levels were expressed as nanograms per gram of wet weight of tissue. All samples were run in duplicate.

### 4.13. Statistical Analysis

GraphPad Prism version 8.01 (GraphPad Prism, San Diego, CA, USA) was used for data analysis. The Shapiro–Wilk normality test was first applied to evaluate the distribution of all quantitative datasets. No criteria were set for including and excluding animals. Since the data showed a normal distribution, a two-way analysis of variance (ANOVA) was conducted, followed by Tukey’s multiple comparison test for post hoc analysis. Correlation analysis was performed using Kendall’s rank correlation τ-b, which is tie-corrected and recommended for the present ordinal and censored data that contain many tied ranks arising both from the floor effects on count variables and from the ceiling (censoring) of latency elapsed [161,167]. Two-sided asymptotic *p*-values were computed. Statistical significance was set at *p* < 0.05. No data points were excluded. The results are presented as mean ± SEM.

## 5. Conclusions

Thus, our study shows that applying the ultrasound stress model to male C57BL/6 mice recapitulates key physiological, behavioral, and hormonal features of clinical fibromyalgia. These features are hyperalgesia, allodynia, ‘timid-like’ behavior, increased HPA activation, decreased levels of blood β-Endorphin and BDNF, and central dysregulation of BDNF receptor TrkB/IL-33 mechanisms, correlating with behavioral abnormalities and pain sensitivity. Second, our study supports the important role of BDNF in the neurobiology of fibromyalgia and suggests, for the first time, the role of the pro-inflammatory cytokine IL-33 in this regulation.

## Figures and Tables

**Table 1:** Shared correlations for CBA, BALB/c, and C57BL/6 mouse strains. ↑—increase in the parameter, ↓—decrease in the parameter, «+»—positive correlation, «−»—negative correlation. RIT—resident–intruder test, SI—social interaction test.

Parameters	CBA	BALB/c	C57BL/6
Tail flick latency and *Trkb* expression in the hippocampus	↑↓ −	↑↓ −	↓↓ +
Latency to attack in RIT and *Trkb* expression in the hippocampus	↓↓ +	↓↓ +	↑↓ −
Latency to attack in RIT and *Trkb* expression in the striatum	↓↓ +	↓↓ +	↑↓ *p* = 0.32
Number of attacks in RIT and *Trkb* expression in the hippocampus	↑↓ −	↑↓ −	↓↓ +
Number of attacks in RIT and *Trkb* expression in the striatum	↑↓ −	↑↓ −	↓↓ *p* = 0.11
Number of attacks in RIT and *Il-33* expression in the amygdala	↑↑ +	↑↑ +	↓↓ +
Duration of attacks in RIT and *Trkb* expression in the hippocampus	↑↓ −	↑↓ −	↓↓ +
Duration of attacks in RIT and *Trkb* expression in the striatum	↑↓ −	↑↓ −	↓↓ +
Duration of attacks in SI and *Bdnf* expression in the hippocampus	↑↑ +	↑↑ +	↓↓ *p* = 0.59

## Data Availability

The original contributions presented in this study are included in the article/Supplementary Materials. Further inquiries can be directed to the corresponding author.

## Supplementary Materials

The following supporting information can be downloaded at: https://www.mdpi.com/article/10.3390/ijms27094051/s1.

## Abbreviations

The following abbreviations are used in this manuscript:

MDD	Major depressive disorder
US	Ultrasound stress
BDNF	Brain-derived neurotrophic factor
TrkB	Tropomyosin receptor kinase B
TrkB-FL	Full-length tropomyosin receptor kinase B
TrkB-T	Truncated tropomyosin receptor kinase B
IL	Interleukin
DSM-5	Diagnostic and Statistical Manual of Mental Disorders, Fifth Edition
SSD	Somatic symptom disorder
HPA	Hypothalamic–pituitary–adrenal
NMDA	N-methyl-D-aspartate receptor
CORT	Corticosterone
PSD95	Postsynaptic density protein 95
PSA-NCAM	Polysialylated neural cell adhesion molecule
TNF	Tumor necrosis factor
MyD88	Myeloid differentiation primary response 88
NF-κB	Nuclear factor kappa B
MAPK	Mitogen-activated protein kinase
RNA	Ribonucleic acid
mRNA	Messenger ribonucleic acid
i.c.v.	Intracerebroventricular
ANOVA	Analysis of variance
SEM	Standard error of the mean
RIT	Resident–intruder test
SI	Social interaction
ARRIVE	Animal Research: Reporting of In Vivo Experiments
RT-qPCR	Reverse transcription quantitative polymerase chain reaction
ELISA	Enzyme-linked immunosorbent assay
cDNA	Complementary DNA
GAPDH	Glyceraldehyde-3-phosphate dehydrogenase
