# Supplementary material for: BDNF and IL-33 Dynamics in an Ultrasound Stress Model of Fibromyalgia-like Phenotypes
Source: Int J Mol Sci. 2026 Apr 30;27(9):4051. doi: 10.3390/ijms27094051 (PMC13163552; doi:10.3390/ijms27094051)
Supplement: Supplementary file 1 [file ijms-27-04051-s001.zip › ijms-4239510-supplementary.pdf]

Supplementary file

Supplementary Table S1. Sequences of the primers used.

Gene	Forward sequence	Reverse sequence
Gapdh	TGCACCACCAACTGCTTAG	GGATGCAGGGATGATGTTC
Bdnf	GGCTGACACTTTTGAGCACGTC	CTCCAAAGGCACTTGACTGCTG
Trkb	GAACATCACGGAAATGATCC	AACTTTCCCGAAGGCTCCTT
Il-33	CTACTGCATGAGACTCCGTTCTG	AGAATCCCGTGGATAGGCAGAG

Supplementary Table S2. Correlations between scores of aggression and pain sensitivity with BDNF, TrkB and IL-33 expression, BDNF, β-Endorphin and glucocorticoids levels in CBA mice. Significant correlations (p<0.05) are in cursive, trends (p<0.1) are underlined, non-significant correlations (p>0.1) are in regular font.

	**Tail flick latency, s**	**Resident-intruder**			**Social interaction**		
		**Latency to attack, s**	**Number of attacks, n**	**Duration of attacks, s**	**Latency to attack, s**	**Number of attacks, n**	**Duration of attacks, s**
**BDNF in the hippocampus**	*p=0.0315*<br>*r=0.5825*	*p=0.0019*<br>*r=-0.7755*	*p=0.0221*<br>*r=0.6135*	*p=0.0025*<br>*r=0.7606*	*p=0.0095*<br>*r=-0.6788*	*p=0.0156*<br>*r=0.6423*	*p=0.0189*<br>*r=0.6275*
**BDNF in the amygdala**	p=0.2676<br>r=0.3167	*p=0.0278*<br>*r=-0.5957*	*p=0.0221*<br>*r=0.5986*	*p=0.0400*<br>*r=0.5603*	*p=0.0145*<br>*r=-0.6480*	*p=0.0479*<br>*r=0.5421*	*p=0.0424*<br>*r=0.5558*
**BDNF in the striatum**	*p=0.0169*<br>*r=0.6357*	*p=0.0146*<br>*r=-0.6496*	*p=0.0127*<br>*r=0.6694*	*p=0.0123*<br>*r=0.6616*	p=0.2057<br>r=-0.3612	p=0.8734<br>r=0.0504	p=0.1246<br>r=0.4329
**TrkB in the hippocampus**	*p=0.0092*<br>*r=-0.6800*	*p=0.0109*<br>*r=0.6721*	*p=0.0020*<br>*r=-0.7719*	*p=0.0019*<br>*r=-0.7741*	*p=0.0155*<br>*r=0.6429*	*p=0.0051*<br>*r=-0.7193*	*p=0.0054*<br>*r=-0.7146*
**TrkB in the amygdala**	p=0.2677<br>r=0.3266	*p=0.0278*<br>*r=-0.5957*	*p=0.0266*<br>*r=0.5986*	*p=0.0071*<br>*r=-0.6999*	*p=0.0189*<br>*r=0.6275*	*p=0.0324*<br>*r=-0.5806*	*p=0.0343*<br>*r=-0.5763*
**TrkB in the striatum**	p=0.0953<br>r=-0.4651	*p=0.0084*<br>*r=0.6901*	*p=0.0046*<br>*r=0.5427*	*p=0.0076*<br>*r=-0.6954*	*p=0.0424*<br>*r=0.5558*	p=0.0671<br>r=-0.5061	p=0.0755<br>r=-0.4943
**IL-33 in the hippocampus**	*p=0.033*<br>*r=0.5781*	p=0.0737<br>r=-0.4968	*p=0.0476*<br>*r=-0.7269*	p=0.0631<br>r=0.5131	*p=0.0361*<br>*r=-0.5712*	*p=0.0195*<br>*r=0.6243*	*p=0.0244*<br>*r=0.6070*
**IL-33 in the amygdala**	*p=0.0221*<br>*r=0.6135*	p=0.2157<br>r=-0.3529	*p=0.0266*<br>*r=0.5986*	p=0.2425<br>r=0.3331	p=0.0937<br>r=-0.4687	p=0.1322<br>r=0.4239	p=0.1294<br>r=0.4277
**IL-33 in the striatum**	*p=0.0476*<br>*r=0.5427*	p=0.3619<br>r=-0.2630	p=0.2629<br>r=0.3195	p=0.3069<br>r=0.2925	p=0.2107<br>r=-0.3814	p=0.1828<br>r=0.3777	p=0.2124<br>r=0.3560
**BDNF in plasma**	*p=0.0323*<br>*r=0.7731*	*p=0.0323*<br>*r=-0.7731*	p=0.0842<br>r=0.6627	p=0.0842<br>r=0.6627	p=0.7421<br>r=-0.1553	p=0.7321<br>r=0.1333	p=0.7321<br>r=0.1333
**β-Endorphin in plasma**	*p=0.0464*<br>*r=0.7382*	p=0.0714<br>r=-0.6872	*p=0.0479*<br>*r=0.7363*	*p=0.0479*<br>*r=0.7363*	p=0.1964<br>r=-0.5175	p=0.1964<br>r=0.5175	p=0.1964<br>r=0.5175
**Corticosterone in plasma**	p=0.1854<br>r=0.4099	p=0.0935<br>r=-0.5090	*p=0.0370*<br>*r=0.6158*	*p=0.0298*<br>*r=0.6371*	*p=0.0305*<br>*r=-0.6324*	*p=0.0195*<br>*r=0.6757*	*p=0.0172*<br>*r=0.6823*
**Cortisol in plasma**	p=0.4827<br>r=0.2928	p=0.0923<br>r=0.-6587	p=0.1223<br>r=0.6099	p=0.1223<br>r=0.6099	p=0.2857<br>r=-0.4520	p=0.1955<br>r=0.5205	p=0.2857<br>r=0.4520

Supplementary Table S3. Correlations between scores of aggression and pain sensitivity with BDNF, TrkB and IL-33 expression, BDNF, β-Endorphin and glucocorticoids levels in BALB/c

mice. Significant correlations (p<0.05) are in cursive, trends (p<0.1) are underlined, non-significant correlations (p>0.1) are in regular font.

	Tail flick latency, s	Resident-intruder			Social interaction		
		Latency to attack, s	Number of attacks, n	Duration of attacks, s	Latency to attack, s	Number of attacks, n	Duration of attacks, s
**BDNF in the hippocampus**	p=0.1246<br>r=0.4311	p=0.7380<br>r=0.0978	p=0.7841<br>r=-0.0803	p=0.9173<br>r=-0.0311	p=0.0831<br>r=-0.4818	p=0.0776<br>r=0.5111	*p=0.0743*<br>*r=0.4936*
**BDNF in the amygdala**	p=0.5716<br>r=-0.1645	p=0.7910<br>r=-0.0777	p=0.7664<br>r=0.0870	p=0.7492<br>r=0.0935	p=0.2209<br>r=0.3496	p=0.2559<br>r=-0.3396	p=0.2299<br>r=-0.3420
**BDNF in the striatum**	p=0.2082<br>r=0.3578	p=0.8029<br>r=-0.0773	p=0.1940<br>r=0.3683	p=0.2127<br>r=0.3542	p=0.7803<br>r=-0.0825	p=0.8734<br>r=0.0504	p=0.7284<br>r=-0.1020
**TrkB in the hippocampus**	*p=0.0389*<br>*r=-0.5623*	*p=0.0489*<br>*r=0.5400*	*p=0.0364*<br>*r=-0.5693*	*p=0.0395*<br>*r=-0.5613*	p=0.1712<br>r=0.3882	p=0.1175<br>r=-0.4573	p=0.1395<br>r=-0.4164
**TrkB in the amygdala**	p=0.0531<br>r=-0.5311	p=0.2728<br>r=0.3134	p=0.1998<br>r=-0.3639	p=0.1921<br>r=-0.3697	p=0.3611<br>r=0.2643	p=0.0991<br>r=-0.4808	p=0.3262<br>r=-0.2840
**TrkB in the striatum**	p=0.5664<br>r=-0.1667	*p=0.0419*<br>*r=0.5556*	*p=0.0044*<br>*r=-0.7278*	*p=0.0053*<br>*r=-0.7172*	p=0.9962<br>r=0.0027	p=0.8900<br>r=0.0437	p=0.9664<br>r=-0.0137
**IL-33 in the hippocampus**	p=0.4100<br>r=0.2378	p=0.3211<br>r=-0.2845	p=0.2886<br>r=0.3036	p=0.3439<br>r=-0.2717	p=0.1097<br>r=-0.4487	p=0.1436<br>r=0.4304	p=0.1062<br>r=0.4523
**IL-33 in the amygdala**	p=0.1967<br>r=0.4511	*p=0.0410*<br>*r=-0.5578*	*p=0.0057*<br>*r=0.7121*	*p=0.0042*<br>*r=0.7306*	p=0.3308<br>r=-0.2808	p=0.3485<br>r=0.2824	p=0.3104<br>r=0.2923
**IL-33 in the striatum**	p=0.0736<br>r=0.4956	p=0.4047<br>r=-0.2400	p=0.1911<br>r=0.3706	p=0.1950<br>r=0.3675	p=0.2097<br>r=-0.3579	p=0.2182<br>r=0.3665	p=0.1941<br>r=0.3695
**BDNF in plasma**	p=0.5318<br>r=-0.2547	p=0.2760<br>r=0.4364	*p=0.0082*<br>*r=-0.8780*	p=0.0169<br>r=-0.8243	p=0.7381<br>r=0.1518	p=0.0655<br>r=-0.7235	p=0.7381<br>r=-0.1518
**β-Endorphin in plasma**	p=0.3253<br>r=0.4038	p=0.5236<br>r=-0.2606	p=0.3262<br>r=0.3963	p=0.2316<br>r=0.4788	p=0.4792<br>r=-0.2899	p=0.1845<br>r=0.5148	p=0.4792<br>r=0.2899
**Corticosterone in plasma**	p=0.1493<br>r=0.4939	p=0.3048<br>r=-0.3620	p=0.3546<br>r=0.3293	p=0.1923<br>r=0.4493	p=0.3048<br>r=-0.3620	p=0.2540<br>r=0.4258	p=0.1750<br>r=0.4698
**Cortisol in plasma**	p=0.1423<br>r=0.5770	p=0.3146<br>r=-0.4072	p=0.0627<br>r=0.6988	p=0.0525<br>r=0.7186	*p=0.0476*<br>*r=0.7306*	p=0.1071<br>r=0.6323	*p=0.0476*<br>*r=0.7638*

Supplementary Table S4. Correlations between scores of aggression and pain sensitivity with BDNF, TrkB and IL-33 expression, BDNF, β-Endorphin and glucocorticoids levels in C57BL/6 mice. Significant correlations (p<0.05) are in cursive, trends (p<0.1) are underlined, non-significant correlations (p>0.1) are in regular font.

	Tail flick latency, s	Resident-intruder			Social interaction		
		Latency to attack, s	Number of attacks, n	Duration of attacks, s	Latency to attack, s	Number of attacks, n	Duration of attacks, s
**BDNF in the hippocampus**	*p=0.0003*<br>*r=0.8464*	p=0.0961<br>r=-0.4653	p=0.0979<br>r=0.4619	p=0.1237<br>r=0.4321	p=0.0623<br>r=-0.5168	p=0.9381<br>r=0.0238	p=0.0751<br>r=0.4985
**BDNF in the amygdala**	*p=0.0029*<br>*r=0.7506*	*p=0.0079*<br>*r=-0.6946*	*p=0.0020*<br>*r=0.7728*	*p=0.0041*<br>*r=0.7336*	p=0.0549<br>r=-0.5291	p=0.9706<br>r=0.0119	*p=0.0467*<br>*r=0.5474*
**BDNF in the striatum**	*p=0.0037*<br>*r=0.7373*	*p=0.0044*<br>*r=-0.7305*	*p=0.0011*<br>*r=0.7975*	*p=0.0035*<br>*r=0.7426*	*p=0.0201*<br>*r=-0.6086*	p=0.7837<br>r=0.0809	*p=0.0165*<br>*r=0.6269*
**TrkB in the hippocampus**	*p=0.0065*<br>*r=0.7038*	*p=0.0079*<br>*r=-0.6946*	*p=0.0070*<br>*r=0.7007*	*p=0.0069*<br>*r=0.7021*	p=0.5440<br>r=-0.1804	p=0.1618<br>r=-0.3903	p=0.4918<br>r=0.2049
**TrkB in the amygdala**	*p=0.0107*<br>*r=0.6704*	*p=0.0079*<br>*r=-0.6842*	*p=0.0032*<br>*r=0.7480*	*p=0.0035*<br>*r=0.7626*	p=0.3114<br>r=-0.2966	p=0.5294<br>r=-0.1833	p=0.3223<br>r=0.2905
**TrkB in the striatum**	p=0.1894<br>r=0.3720	p=0.3210<br>r=-0.2855	p=0.1052<br>r=0.4528	p=0.1133<br>r=0.4433	p=0.6813<br>r=-0.1254	p=0.5026<br>r=-0.1952	p=0.5064<br>r=0.1988
**IL-33 in the hippocampus**	*p=0.0193*<br>*r=0.6259*	*p=0.0023*<br>*r=-0.7665*	*p=0.0039*<br>*r=0.7367*	*p=0.0090*<br>*r=0.6841*	p=0.2005<br>r=-0.3700	p=0.6311<br>r=-0.1404	p=0.2216<br>r=0.3517
**IL-33 in the**	*p=0.0002*	p=0.0961	*p=0.0020*	*p=0.0024*	*p=0.0137*	p=0.7462	*p=0.0147*

**amygdala**	*r=0.8620*	r=-0.4653	*r=0.7728*	*r=0.7629*	*r=-0.6392*	r=0.0952	*r=0.6330*
**IL-33 in the striatum**	*p=0.0051*<br>*r=0.7194*	*p=0.0005*<br>*r=-0.8294*	*p=0.0022*<br>*r=0.7628*	*p=0.0043*<br>*r=0.7314*	p=0.1181<br>r=-0.4484	p=0.5026<br>r=-0.1952	p=0.5064<br>r=0.1988
**BDNF in plasma**	p=0.1738<br>r=0.5295	p=0.4006<br>r=-0.3436	p=0.1712<br>r=0.5372	p=0.1973<br>r=0.5154	p=0.4286<br>r=-0.3528	p=0.8595<br>r=0.0706	p=0.4286<br>r=0.3528
**β-Endorphin in plasma**	*p=0.0464*<br>*r=0.7382*	p=0.0976<br>r=-0.6420	*p=0.0211*<br>*r=0.8138*	*p=0.0286*<br>*r=0.7902*	p=0.2679<br>r=-0.4654	p=0.8988<br>r=0.0581	p=0.2679<br>r=0.4654
**Corticosterone in plasma**	*p=0.0043*<br>*r=-0.7532*	*p=0.0123*<br>*r=0.6797*	*p=0.0209*<br>*r=-0.6425*	*p=0.0173*<br>*r=-0.6584*	p=0.2972<br>r=0.3158	p=0.4143<br>r=0.2469	p=0.3077<br>r=-0.3084
**Cortisol in plasma**	p=0.0619<br>r=-0.7103	*p=0.0054*<br>*r=0.9027*	p=0.0054<br>r=-0.8961	*p=0.0027*<br>*r=-0.9271*	p=0.5357<br>r=0.2650	p=0.4810<br>r=0.2937	p=0.5357<br>r=-0.2650
